# eMERGE Phenome-Wide Association Study (PheWAS) identifies clinical associations and pleiotropy for stop-gain variants

**DOI:** 10.1186/s12920-016-0191-8

**Published:** 2016-08-12

**Authors:** Anurag Verma, Shefali S. Verma, Sarah A. Pendergrass, Dana C. Crawford, David R. Crosslin, Helena Kuivaniemi, William S. Bush, Yuki Bradford, Iftikhar Kullo, Suzette J. Bielinski, Rongling Li, Joshua C. Denny, Peggy Peissig, Scott Hebbring, Mariza De Andrade, Marylyn D. Ritchie, Gerard Tromp

**Affiliations:** 1 Department of Biochemistry and Molecular Biology, Center for Systems Genomics, Pennsylvania State University, University Park, PA USA; 2 Biomedical and Translational Informatics, Geisinger Health System, Danville, PA USA; 3 Division of Molecular Biology and Human Genetics, Department of Biomedical Sciences, Faculty of Medicine and Health Sciences, Stellenbosch University, Tygerberg, 7505 South Africa; 4 Case Western Reserve University, Cleveland, OH USA; 5 Department of Medicine, Division of Medical Genetics, University of Washington, Seattle, WA USA; 6 Vanderbilt University, Nashville, TN USA; 7 Marshfield Clinic, Marshfield, WI USA; 8 Mayo Clinic, Rochester, MN USA; 9 National Human Genome Research Institute, Bethesda, MD USA

## Abstract

**Background:**

We explored premature stop-gain variants to test the hypothesis that variants, which are likely to have a consequence on protein structure and function, will reveal important insights with respect to the phenotypes associated with them. We performed a phenome-wide association study (PheWAS) exploring the association between a selected list of functional stop-gain genetic variants (variation resulting in truncated proteins or in nonsense-mediated decay) and an extensive group of diagnoses to identify novel associations and uncover potential pleiotropy.

**Results:**

In this study, we selected 25 stop-gain variants: 5 stop-gain variants with previously reported phenotypic associations, and a set of 20 putative stop-gain variants identified using dbSNP. For the PheWAS, we used data from the electronic MEdical Records and GEnomics (eMERGE) Network across 9 sites with a total of 41,057 unrelated patients. We divided all these samples into two datasets by equal proportion of eMERGE site, sex, race, and genotyping platform. We calculated single effect associations between these 25 stop-gain variants and ICD-9 defined case-control diagnoses. We also performed stratified analyses for samples of European and African ancestry. Associations were adjusted for sex, site, genotyping platform and the first three principal components to account for global ancestry. We identified previously known associations, such as variants in LPL associated with hyperglyceridemia indicating that our approach was robust. We also found a total of three significant associations with p < 0.01 in both datasets, with the most significant replicating result being LPL SNP rs328 and ICD-9 code 272.1 “Disorder of Lipoid metabolism” (p_discovery_ = 2.59x10-6, p_replicating_ = 2.7x10-4). The other two significant replicated associations identified by this study are: variant rs1137617 in KCNH2 gene associated with ICD-9 code category 244 “Acquired Hypothyroidism” (p_discovery_ = 5.31x103, p_replicating_ = 1.15x10-3) and variant rs12060879 in DPT gene associated with ICD-9 code category 996 “Complications peculiar to certain specified procedures” (p_discovery_ = 8.65x103, p_replicating_ = 4.16x10-3).

**Conclusion:**

In conclusion, this PheWAS revealed novel associations of stop-gained variants with interesting phenotypes (ICD-9 codes) along with pleiotropic effects.

**Electronic supplementary material:**

The online version of this article (doi:10.1186/s12920-016-0191-8) contains supplementary material, which is available to authorized users.

## Background

Genetic variations can result in changes in the success of transcription and translation, as well as modification of the structure and function of resulting proteins. These changes are also responsible for potential downstream effects across pathways and ultimately affecting phenotypic outcomes. Thus, exploring the associations between functional genetic variants and a number of phenotypes can be helpful in highlighting the impact of genetic architecture on outcomes in a more biologically interpretable manner. A number of resources for identifying the function of genetic variants on transcription, translation, protein structure and function have emerged, providing a way to highlight genetic variants that likely have an impact on protein structure or function. Loss-of-function or gain-of-function variants are responsible for changing the function of protein products, and these functional variants have been shown to be important for identifying clinically relevant associations in pharmacogenetic studies [1, 2]. Nonsense mutations result in premature termination of translation that result in the production of in non-functional polypeptides [3]. Variations that result in new stop-codon are referred to as stop-gain variants. Stop-gain variants have been shown to be associated with Mendelian diseases in the OMIM database [4]. Thus, there is the potential for stop-gain variants to explain stronger effects than other types of variants [5] In this study, we applied an approach to explore phenotypes conditional on genotypes, namely phenome wide association study (PheWAS).

PheWAS evaluates associations between selected genetic variants and an extensive set of phenotypes and thus is an effective approach. This approach has been successfully used to identify disease associations using EHR (Electronic Health Record) -based phenotype data [6, 7]. PheWAS has also been implemented within epidemiological and clinical trials datasets and has become an important tool for identifying novel associations as well as discovering pleiotropic effects [8–11]. PheWAS can identify associations across multiple phenotypes, where genetic variation is associated with more than one phenotype, some of which may be due to pleiotropy [12] and also some that are observed through multiple GWA studies can thus be identified with this approach. An example includes variation in the human leukocyte antigen (HLA) region known to be associated with variety of autoimmune diseases [13]. PheWAS has been shown to be effective at identifying cross-phenotype associations (pleiotropic associations) of functional variants [14]. In this study, we hypothesized that stop-gain variants are more likely to impact clinically relevant outcomes compared with the common variants targeted by genome-wide genotyping arrays. Therefore, to identify associations between EHR-based phenotypes and stop-gain variants, we performed a PheWAS between 25 selected stop-gain variants and multiple phenotypes in EHR data and to determine whether one or more of these putative functional variants are associated with any clinical conditions. Unlike GWAS studies where the clinical relevance of identified variants is difficult to explain, with this study we aimed at study only clinical or scientific relevant variants and its association with a comprehensive list of ICD-9 diagnoses codes. The Electronic MEdical Records and GEnomics (eMERGE) is a large dataset consisting of many sites where samples are also genotyped on various platforms. We provide a first of its kind approach to describe methods and challenges in investigating samples from various demographic regions within USA.

## Methods

### Study dataset

For the study we used the imputed genotype data available from the electronic medical records and genomics (eMERGE) network [15]. The eMERGE Network consists of 9 sites that are aimed at identifying genotype associations using phenotype data from the EHR [16]. The eMERGE Network consists of 55,289 samples genotyped across multiple platforms and imputed to 1000 Genomes reference panel covering ~18 million variants with age of participants ranging from infants to above 90 years of age. In order to identify relevant associations and replications, we used 41,057 adult samples (≥19 years of age) from the eMERGE Network. EHRs contain a variety of kinds of data, including International Classification of Diseases, Ninth Revision (ICD-9) codes, clinical lab variables, medication, demographics etc. ICD-9 codes classify variety of signs, symptoms, diseases, and injuries. In this study, we used ICD-9 diagnosis codes to define case/control status for a variety of conditions. Since samples in eMERGE were genotyped on several different platforms, genotypic imputation was performed on these datasets to combine them. eMERGE data were imputed using IMPUTE2 [17] with phasing done using SHAPEIT2 [16].

### Discovery and replication dataset

To obtain highly robust results from this PheWAS, we divided the eMERGE dataset into a discovery and replication set using a random sampling approach. Samples in eMERGE are from diverse populations and several genotyping platforms were used in the analysis. Therefore, in order to consider confounding factors when dividing the data, we used a stratified sampling strategy to reduce the impact of potential biases that could arise after dividing the data due to extreme diversity in the dataset. We proportionately allocated samples by each stratum; where stratum is a class to which samples were distributed by sex, eMERGE site, genotyping platform, and race/ethnicity. We had a total of 21,085 samples in the discovery set and 21,065 samples in the replication dataset. Additional file 1: Table S1 shows the distribution of samples across each dataset by each stratum before quality control was performed on samples.

### Quality control of genetic data

We used samples from eMERGE phase I and II. Samples from all sites in eMERGE I were genotyped on one of two platforms (Illumina 660 and 1 M) and in eMERGE-II, samples from all sites (9 sites) were genotyped on different platforms (a total of 9 different platforms) [15, 18, 19]. The overlap among the SNPs from different genotyping platforms was fairly small (about 20,000 SNPs), thus each dataset was imputed to enable robust combination of datasets. Described in detail in a previous publication, we imputed all samples using IMPUTE2 best practices guidelines and 1000 Genomes reference panel [20] which resulted in approximately 38 million variants across the entire dataset. Identity by decent (IBD) estimation was performed using PLINK’s method of moment in R package SNPrelate [21] in order identify and remove related samples from further analysis. The evaluation of relatedness was more appropriate after dividing the eMERGE dataset because there are known sample relationships from some sites and IBD estimation after randomly dividing the dataset dropped fewer samples. One member from a pair of individuals with kinship coefficient > 0.125 was removed which resulted in 20,526 and 20,531 unrelated samples in discovery and replication datasets respectively. Principal Component Analysis (PCA) was performed using smartpca program in Eigensoft package [22]. The first three principal components or eigenvectors were then used to adjust models for global ancestry.

### Identifying stop-gain variants

To determine the functional impact of the all variants, we first annotated all SNPs that passed QC criteria with six bioinformatics annotation and prediction tools i.e., SNPeff [23], ANNOVAR [24], GEMINI [25], Variant effect predictor [26], VAT [27] and SeattleSeq [28]. The results of variant function predicted across these tools were quite different for the SNPs of this study as shown in Additional file 2: Table S2. As observed by others, functional prediction tools differ in their predictions [29]. To obtain a more robust measure of functionality of these eMERGE SNPs, we thus queried all genetic variants of this study against all stop-gain variants found in dbSNP137 and identified 225 stop-gain variants in our data. We then compared these dbSNP-annotated variants with the cross tissue average of every transcript generated from Illumina BodyMap 2.0 project data [30]. Within the Illumina BodyMap project there are 16 different tissues with RNAseq data and we considered the most widely expressed transcript as the most canonical transcript. After this filtering step, a total of 46 likely loss-of-function stop-gain variants were selected. To serve as positive controls, we included 9 additional SNPs with known association with traits. The list of 46 likely stop-gain variants and 9 proof-of-principle SNPs, were then extracted from both discovery and replication datasets. Variants below minor allele frequency (MAF) threshold of 0.005 were filtered out, thus resulting in total of 25 variants that were considered for association testing. Out of the 25 variants selected, 20 were identified through the annotation pipeline as stop-gain variants and five variants (Table 1) were used as positive controls for proof-of-concept validation.

**Table 1:** Proof of concept null variants. Note that ICD-9 codes are not shown for all traits because there is not a known association with an ICD-9 code for all traits

SNP	Gene	^a^Trait Previously Associated
rs328	*LPL*	Pure hyperglyceridemia (ICD9 272.1) [14]
rs2814778	*DARC*	White Blood Cell count [47]
rs1815739	*ACTN3*	Dystrophinopathy [48], type 2 diabetes [49]
rs16910526	*CLEC7A*	Inflammatory bowel disease [50], candidiasis [51], aspergillosis [52]
rs601338	*FUT2*	Gastroenteritis [53], Crohn’s disease [53]

^a^ICD-9 codes are not shown for all traits because there is not a known association with an ICD-9 code based definition of the condition

### Phenotype data

The phenotypic data consisted of 11,879 distinct ICD-9 codes for 41,057 individuals with genotype data. We defined case-control status for each ICD-9 code, where a case status is assigned when an individual has ≥ 3 instances of an ICD-9 code and control status is assigned based on the absence of an ICD-9 code. In cases of samples with more than one but fewer than three ICD-9 code instances, we removed them from analysis for that ICD-9 code. We further excluded the ICD-9 diagnoses that were present in fewer than 10 individuals. Using these filtering criteria on ICD-9 code data, there were 20,526 samples and 2,879 ICD-9 codes in the discovery dataset and 20,531 samples and 2,854 distinct ICD-9 codes in the replication dataset.

### Association testing

We conducted standard and penalized regression in the discovery and replication dataset separately using PLATO (http://www.ritchielab.psu.edu/software/plato-download) and we adjusted the models for sex, site, platform, and the first three principal components to account for global ancestry. In the discovery dataset, we performed association testing with penalized logistic regression between 25 SNPs and 2,859 ICD-9 based case/control status and in the replication dataset 2,854 ICD-9 codes were included. Results of tests of association were visualized using Synthesis-View [31]. Because some of the SNPs might have shown association in one racial/ancestry group compared to another, and one of our proof-of-concept SNPs is more prevalent in people of African ancestry (*DARC* variant rs2814778) we also performed association testing stratified by the two largest racial/ethnic groups in the present study: European ancestry (EA) and African ancestry (AA).

**Fig. 1:**
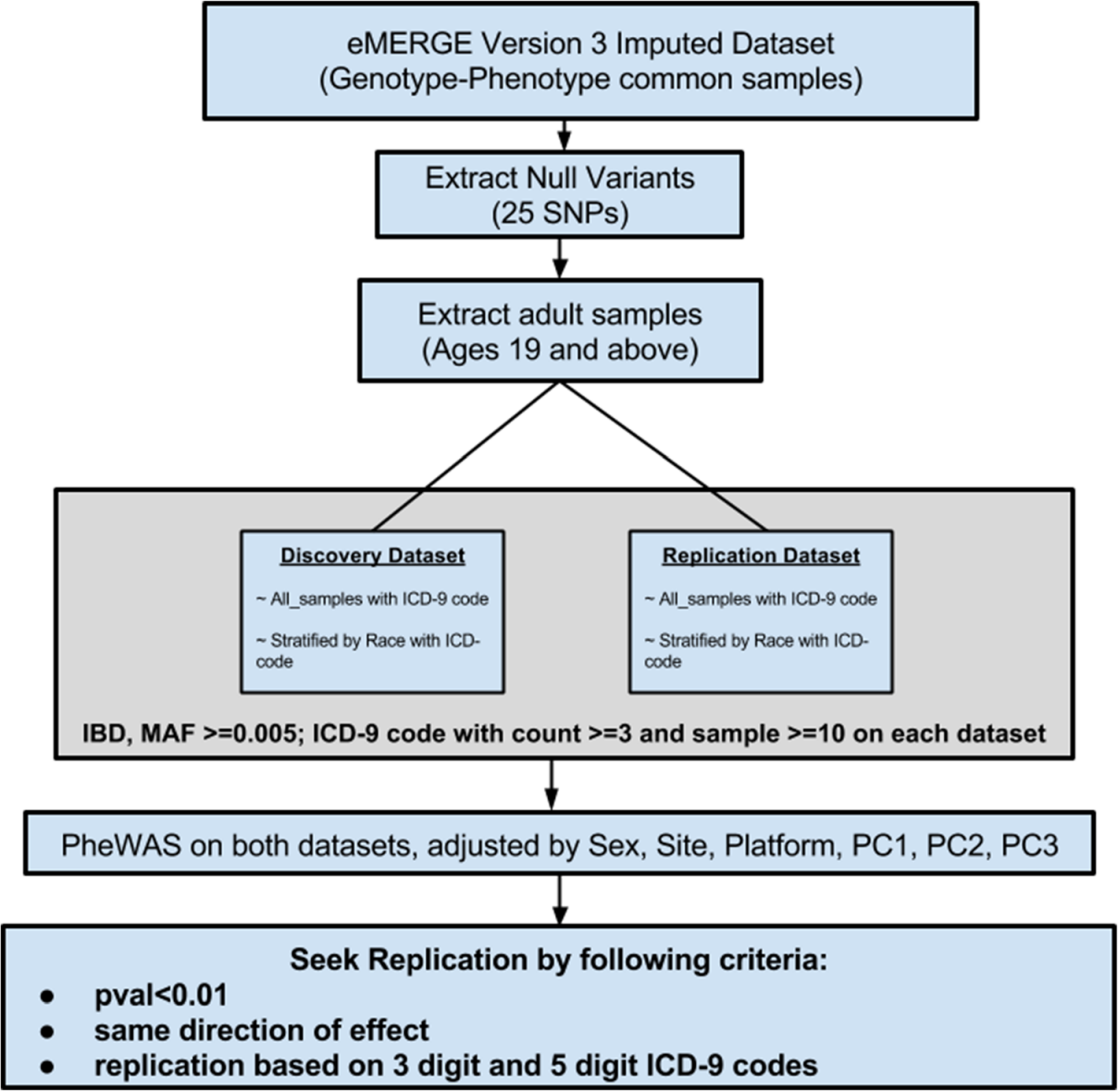
Flowchart showing the analysis pipeline for PheWAS on functional variants in eMERGE data

ICD-9 codes classify diagnoses; there are three digit ICD-9 codes that specify disease categories (e.g. code 405 for “secondary hypertension”) that can are further be further subdivided using multiple four or five digit sub- ICD-9 codes (e.g. 405.1 for “benign secondary hypertension”, 405.11 “benign renovascular hypertension”). We therefore analyzed results based on replication requiring the exact ICD-9 code for more specific replication (three to five digit sub- ICD-9 codes), as well as evaluating results based on replication requiring only the same three digit ICD-9 code category, a more broad replication for a given case/control diagnosis. For seeking replication, we required a *P* < 0.01 with the same direction of genetic effect in both the testing discovery and replication dataset for the same SNP, and the same 3-digit ICD-9 code category as well as the exact same code (from three digit to 5 digit). Complete pipeline of the process from selecting of variants to running association analysis is shown in Fig. 1.

## Results

Association analyses for the discovery and replication datasets were performed independently. We performed both standardized and penalized regression analyses, however, standard regression failed due to complete or quasi-complete separation. Therefore, we describe results from only penalized logistic regression in the sections below. On the individual dataset level we identified 192 SNP-diagnoses associations in the discovery set and 195 SNP-diagnosis associations in replication dataset with a *p* < 0.01, at the 5-digit level ICD-9 code level (Additional file 3: Table S3 and Additional file 4: Table S4).

The most significant association in the discovery dataset was the association between ICD-9 coded 272.1 “Pure hyperglyceridemia” and the proof-of-principle *LPL* SNP rs328 (P = 2.59 × 10^-6^, OR = 0.52 [95 % CI: 0.39, 0.70]), replicating a previously published association for this SNP. The most significant association in the replication dataset was between the *S*NP rs601338 in *CC2D2A* gene with ICD-9 code 266.2 “B-complex deficiencies” with P = 3.73 × 10^-5^, OR = 0.74 [0.64, 0.85]. SNP rs601338 is in high LD with a non-synonymous common variant rs602662 which has a known association with plasma vitamin B12 [32].

We sought replication of results between the discovery and replication datasets at the three-digit level (more broad ICD-9 code level) as well as for the exact ICD-9 code (anywhere from the exact 3 digit to 5 digit code for a given association).

**Fig. 2:**
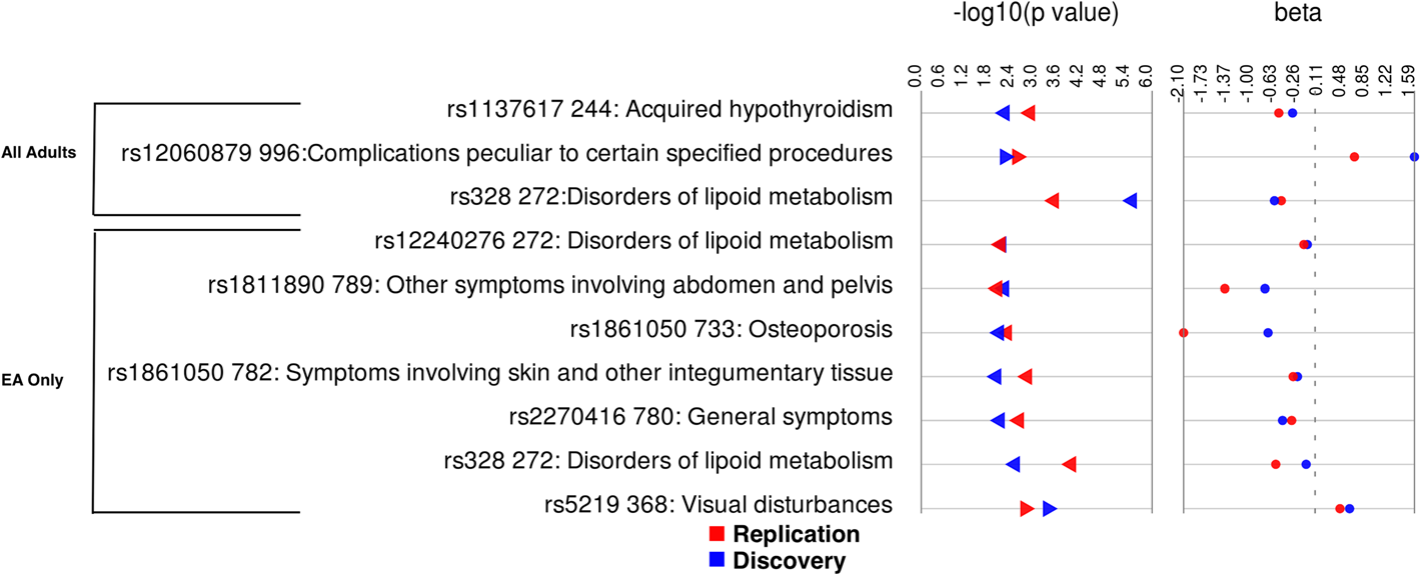
Synthesis-view plot for all replicating results based on 3 digit ICD-9 code category defined conditions from adults and EA for all adults tested and for European American (EA) adults analysis. Labels on the y-axis are SNP-ICD9 code category –Description of ICD9 code. This plot shows p-value in both datasets (discovery dataset in blue and replicating dataset in red) and the direction of triangle represents the direction of genetic effect of the association

Only one association was found replicating at the 5-digit “exact” ICD-9 code level. A total of three associations replicated at the broader 3 digit ICD-9 level with *P* < 0.01 and same direction of genetic effect (Fig. 2).

We also included proof-of-principle variants that have known association with disease, and evaluated how well we replicated known associations for these variants. As previously mentioned, the most significant result in the discovery dataset was for *LPL* SNP rs328, and ICD9 code 272.1. This result replicated with P = 2.7 × 10^-4^. The SNP rs328 is a premature stop codon in gene *LPL* (lipoprotein lipase) known to be associated with lipid metabolism [33, 34]. In analyses including all adults, we did not find replication for any other proof-of-principle SNPs that were included in our list of variants. Among the novel results consistently associated in both the discovery and replication datasets at the 3-digit level was a variant in *KCNH2* (rs1137617) a gene known to cause long QT syndrome with ICD-9 codes 244 “Acquired hypothyroidism” (P_discovery_ = 5.31x 10^-3^ and P_replication_ = 1.15x10^-3^).

Stratified analyses among European-American adults resulted in seven associations replicating by the 3 digit ICD-9 code category criteria (Fig. 1). Among the top associations was *KCNJ11* SNP rs5219 and ICD-9 368 “Visual Disturbances” with discovery P = 6.6 × 10^-4^ and replication P =2.5 × 10^-3^. Interestingly, we observed that majority of the samples that were case for ICD-9 code 368 also had diagnosis for Diabetes Mellitus (ICD-9 code category 250). *KCNJ11* rs5219 is known to be associated with Type 2 diabetes [35–37], thus suggesting a potential interesting link between visual disturbances and diabetes. We found the association between *LPL* rs328 and disorders of lipid metabolism again in the European American adult analyses for both discovery and replication datasets (P = 2.8 × 10^-3^ and 9.91 × 10^-5^, respectively). We did not find any statistically significant associations replicating for the African Americans adult analyses.

## Discussion

In this study, we focused on examining the effect of stop-gain variants on disease using a PheWAS approach. Stop-gain variants were selected for this analysis because these are high impact variants and they are expected to be clinically relevant [38, 39]. PheWAS has been proven as an effective approach in identifying already known associations as well as novel associations. We identified three novel associations along with replicating an already known association between a variant in *LPL* gene (rs328) and pure hyperglycedemia [33].

As mentioned above, we calculated associations by performing both standardized as well as penalized logistic regression in both discovery and replication dataset separately using PLATO after adjusting the models for sex, site, platform and the first 3 principal components. The reason behind conducting analyses by two methods is that none of the models from standardized regression converged. Non-convergence is an issue in logistic regression that occurs when likelihood maximization algorithm fails and the estimates from such regression are questionable [40]. The cause of non-convergence is due to certain data patterns leading to complete or quasi-complete separation [40] i.e. when the outcome variable completely separates the predictor variable. As described, the merged imputed data consisted of multiple sites and platforms. Therefore, we adjusted our regression model by site and platform to account for any confounding biases. Upon further investigation, we found that many ICD-9 codes had either “0” cases or “0” controls for one or more categories (site and platform), causing quasi-complete separation. A penalized maximum likelihood estimation approach proposed by David Firth allows solving separation problem and provides converged model [41]. Thus, we implemented Firth regression into PLATO and repeated the association testing with this new-method.

For our novel results meeting our criteria for replication across the two datasets with a match on ICD-9 codes category, we identified a novel association between *KCNH2* SNP rs1137617 and acquired hypothyroidism (ICD-9 code 244). The *KCNH2* gene is known to be associated with long QT syndrome. Prolonged QT intervals are also known to be affected due to thyroid stimulating hormone and it has also been observed that patients with hypothyroidism show higher QT dispersions [42, 43]. Thus, this association between *KCNH2* and hypothyroidism is of potential interest.

In European Americans we identified seven associations that replicated. Among these, replicating results, we found potentially pleiotropic associations for SNP rs1861050 in *CC2D2A* gene and the diagnoses of osteoporosis and edema.

Another interesting association was between *KCNJ11* gene and visual disturbances. *KCJN11* gene is critical in regulation of insulin and is known to be associated with type 2 diabetes mellitus [44, 45]. Genome-wide association studies have found polymorphism in *KCNJ11* (rs5219) to be associated with diabetic retinopathy [46] which may be related to the visual disturbances association identified in the current study.

PheWAS is a method to generate hypotheses by testing a selected set of SNPs and many phenotypes and thus there remains the challenge of correcting for multiple testing. One way to correct for multiple tests is by using Bonferroni correction; however, this is often not appropriate in PheWAS due to the non-independence of the SNPs as well as the phenotypes being tested. In order to address the challenge of multiple testing corrections without using a Bonferroni correction, we instead sought replication of associations by dividing the data into two independent dataset to identify consistent and replicating associations. A limitation of this study was the selection of only 25 null variants. An additional limitation is the selection of only ICD-9 codes. It is possible that with richer, more robust phenotypes, many novel associations would be identified.

Even with these shortcomings, this study design and analysis strategy may provide a more comprehensive exploration of the clinical relevance of known “functional” elements in the genome. Future work should include a substantial expansion of functional variants of interest, based on both protein coding and gene regulation relevance.

## Additional files

Additional file 1: Table S1. Distribution of samples across the two datasets. (XLSX 44 kb) Additional file 2: Table S2. Annotation of 20 variants (excluding 5 proof of principle variants) from different prediction tools. (XLSX 55 kb) Additional file 3: Table S3. Dataset 1 results at *p*-value significance of 0.01. (XLSX 67 kb) Additional file 4: Table S4. Dataset 2 results at *p*-value significance of 0.01. (XLSX 58 kb)

## References

[1] Grant RW, Wexler DJ (2010). Loss-of-function CYP2C9 variants: finding the correct clinical role for Type 2 diabetes pharmacogenetic testing. Expert Rev Cardiovasc Ther.

[2] Scott SA (2011). Personalizing medicine with clinical pharmacogenetics. Genet Med.

[3] Kervestin S, Jacobson A (2012). NMD: a multifaceted response to premature translational termination. Nat Rev Mol Cell Biol.

[4] Rausell A, et al. Analysis of Stop-Gain and Frameshift Variants in Human Innate Immunity Genes. PLoS Comput Biol 2014;10:e1003757. doi:10.1371/journal.pcbi.1003757. 10.1371/journal.pcbi.1003757 PMC4110073 25058640

[5] Chen R, Davydov EV, Sirota M, Butte AJ (2010). Non-synonymous and synonymous coding SNPs show similar likelihood and effect size of human disease association. PLoS One.

[6] Namjou B (2014). Phenome-wide association study (PheWAS) in EMR-linked pediatric cohorts, genetically links PLCL1 to speech language development and IL5-IL13 to Eosinophilic Esophagitis. Front Genet.

[7] Denny JC (2013). Systematic comparison of phenome-wide association study of electronic medical record data and genome-wide association study data. Nat Biotechnol.

[8] Denny JC (2010). PheWAS: demonstrating the feasibility of a phenome-wide scan to discover gene-disease associations. Bioinformatics.

[9] Pendergrass SA (2013). Phenome-wide association study (PheWAS) for detection of pleiotropy within the Population Architecture using Genomics and Epidemiology (PAGE) Network. PLoS Genet.

[10] Hebbring SJ (2014). The challenges, advantages and future of phenome-wide association studies. Immunology.

[11] Hall MA (2014). Detection of pleiotropy through a Phenome-wide association study (PheWAS) of epidemiologic data as part of the Environmental Architecture for Genes Linked to Environment (EAGLE) study. PLoS Genet.

[12] Solovieff N, Cotsapas C, Lee PH, Purcell SM, Smoller JW (2013). Pleiotropy in complex traits: challenges and strategies. Nat Rev Genet.

[13] Gough SC, Simmonds M (2007). The HLA Region and Autoimmune Disease: Associations and Mechanisms of Action. Curr Genomics.

[14] Ye Z (2015). Phenome-wide association studies (PheWASs) for functional variants. Eur J Hum Genet.

[15] Gottesman O. et al. The Electronic Medical Records and Genomics (eMERGE) Network: past, present, and future. Genet Med 2013. doi:10.1038/gim.2013.72 10.1038/gim.2013.72 PMC3795928 23743551

[16] Howie B, Fuchsberger C, Stephens M, Marchini J, Abecasis GR (2012). Fast and accurate genotype imputation in genome-wide association studies through pre-phasing. Nat Genet.

[17] Howie BN, Donnelly P, Marchini J (2009). A flexible and accurate genotype imputation method for the next generation of genome-wide association studies. PLoS Genet.

[18] McCarty CA (2011). The eMERGE Network: A consortium of biorepositories linked to electronic medical records data for conducting genomic studies. BMC Med Genomics.

[19] Khoury MJ, Millikan R, Little J, Gwinn M (2004). The emergence of epidemiology in the genomics age. Int J Epidemiol.

[20] Verma SS (2014). Imputation and quality control steps for combining multiple genome-wide datasets. Front Genet.

[21] Zheng X, et al. A High-performance Computing Toolset for Relatedness and Principal Component Analysis of SNP Data. Bioinformatics bts606 2012. doi:10.1093/bioinformatics/bts606 10.1093/bioinformatics/bts606 PMC3519454 23060615

[22] Price AL (2006). Principal components analysis corrects for stratification in genome-wide association studies. Nat Genet.

[23] Cingolani P (2012). A program for annotating and predicting the effects of single nucleotide polymorphisms, SnpEff: SNPs in the genome of Drosophila melanogaster strain w1118; iso-2; iso-3. Fly (Austin).

[24] Wang K, Li M, Hakonarson H (2010). ANNOVAR: functional annotation of genetic variants from high-throughput sequencing data. Nucl Acids Res.

[25] Paila U, Chapman BA, Kirchner R & Quinlan AR. GEMINI: Integrative Exploration of Genetic Variation and Genome Annotations. PLoS Comput Biol 2013;9. 10.1371/journal.pcbi.1003153 PMC3715403 23874191

[26] McLaren W (2010). Deriving the consequences of genomic variants with the Ensembl API and SNP Effect Predictor. Bioinformatics.

[27] Habegger L (2012). VAT: a computational framework to functionally annotate variants in personal genomes within a cloud-computing environment. Bioinformatics.

[28] Ng SB (2009). Targeted capture and massively parallel sequencing of 12 human exomes. Nature.

[29] McCarthy DJ (2014). Choice of transcripts and software has a large effect on variant annotation. Genome Medicine.

[30] Tonner P, Srinivasasainagendra V, Zhang S, Zhi D (2012). Detecting transcription of ribosomal protein pseudogenes in diverse human tissues from RNA-seq data. BMC Genomics.

[31] Pendergrass SA, Dudek SM, Crawford DC, Ritchie MD (2010). Synthesis-View: visualization and interpretation of SNP association results for multi-cohort, multi-phenotype data and meta-analysis. BioData Min.

[32] Hazra A (2008). Common variants of FUT2 are associated with plasma vitamin B12 levels. Nat Genet.

[33] Webster RJ (2009). The association of common genetic variants in the APOA5, LPL and GCK genes with longitudinal changes in metabolic and cardiovascular traits. Diabetologia.

[34] Welter D (2014). The NHGRI GWAS Catalog, a curated resource of SNP-trait associations. Nucl Acids Res.

[35] Scott LJ (2007). A genome-wide association study of type 2 diabetes in Finns detects multiple susceptibility variants. Science.

[36] Diabetes Genetics Initiative of Broad Institute of Harvard and MIT, Lund University, Novartis Institutes of BioMedical Research (2007). Genome-wide association analysis identifies loci for type 2 diabetes and triglyceride levels. Science.

[37] Timpson NJ (2009). Adiposity-related heterogeneity in patterns of type 2 diabetes susceptibility observed in genome-wide association data. Diabetes.

[38] Stenson PD (2009). The Human Gene Mutation Database: 2008 update. Genome Medicine.

[39] Consortium, T. 1000 G. P (2010). A map of human genome variation from population-scale sequencing. Nature.

[40] Allison P. Convergence Faliure in logistic Regression. at <http://www2.sas.com/proceedings/forum2008/360-2008.pdf>

[41] Firth D (1993). Bias reduction of maximum likelihood estimates. Biometrika.

[42] Galetta F (2008). Changes in heart rate variability and QT dispersion in patients with overt hypothyroidism. Eur J Endocrinol.

[43] Bakiner O (2008). Subclinical hypothyroidism is characterized by increased QT interval dispersion among women. Med Princ Pract.

[44] Bell GI, Polonsky KS (2001). Diabetes mellitus and genetically programmed defects in beta-cell function. Nature.

[45] Florez JC (2007). Type 2 diabetes-associated missense polymorphisms KCNJ11 E23K and ABCC8 A1369S influence progression to diabetes and response to interventions in the Diabetes Prevention Program. Diabetes.

[46] Liu N-J (2015). An analysis of the association between a polymorphism of KCNJ11 and diabetic retinopathy in a Chinese Han population. Eur J Med Res.

[47] Reiner AP (2011). Genome-wide association study of white blood cell count in 16,388 African Americans: the continental origins and genetic epidemiology network (COGENT). PLoS Genet.

[48] Suminaga R, Matsuo M, Takeshima Y, Nakamura H, Wada H (2000). Nonsense mutation of the alpha-actinin-3 gene is not associated with dystrophinopathy. Am J Med Genet.

[49] Riedl I, Osler ME, Benziane B, Chibalin AV & Zierath JR. Association of the ACTN3 R577X polymorphism with glucose tolerance and gene expression of sarcomeric proteins in human skeletal muscle. Physiol Rep 2015;3(3). doi:10.14814/phy2.12314. 10.14814/phy2.12314 PMC4393151 25780092

[50] Moyes DL, Naglik JR (2012). The mycobiome: influencing IBD severity. Cell Host Microbe.

[51] Ferwerda B (2009). Human dectin-1 deficiency and mucocutaneous fungal infections. N Engl J Med.

[52] Carvalho A (2008). Polymorphisms in toll-like receptor genes and susceptibility to pulmonary aspergillosis. J Infect Dis.

[53] McGovern DPB (2010). Fucosyltransferase 2 (FUT2) non-secretor status is associated with Crohn’s disease. Hum Mol Genet.

